# C-reactive protein and the neutrophil-to-lymphocyte ratio on admission predicting bacteraemia with COVID-19

**DOI:** 10.1080/07853890.2023.2278618

**Published:** 2023-11-08

**Authors:** Yoshitaka Ueda, Naoto Yokogawa, Kengo Murata, Tetsuya Kashiyama

**Affiliations:** a Department of Rheumatic Diseases, Tokyo Metropolitan Tama Medical Center, Tokyo, Japan; b Department of Emergency and General Medicine, Tokyo Metropolitan Tama Medical Center, Tokyo, Japan; c Department of Emergency and Critical Care, Tokyo Metropolitan Tama Medical Center, Tokyo, Japan

**Keywords:** covid-19, bacteraemia, co-infection, cRP, nLR, inflammation

## Abstract

**Background:**

Bacteraemia can co-occur with COVID-19. The present study aimed to determine the cut-off value for C-reactive protein (CRP) and the neutrophil-to-lymphocyte ratio (NLR) for predicting bacteraemia in patients with COVID-19.

**Methods:**

Patients admitted to Tokyo Metropolitan Tama Medical Centre for COVID-19 treatment between 1 April 2020 and 30 October 2022 were included. Patients transferred from other hospitals and those whose CRP and/or neutrophil count was not measured at admission were excluded. Community-acquired bacteraemia was diagnosed if true bacteraemia was diagnosed in patients *via* a blood culture performed within 72 h of admission. The cut-off value for CRP and the NLR for community-acquired bacteraemia were determined using receiver operating characteristic analysis.

**Results:**

Among 2989 patients hospitalized for COVID-19 treatment, 19 received the diagnosis of community-acquired bacteraemia, for which CRP ≥ 6.3 was determined to be the cut-off value. The sensitivity and specificity of the cut-off was 89.5% and 73.3%, respectively. The NLR cut-off value was ≥ 7.7, which had a sensitivity and specificity of 84.2% and 84.0%, respectively.

**Conclusions:**

Considering the possibility of the co-occurrence of bacteraemia with COVID-19, a blood culture should be performed when CRP is ≥ 6.3 or the NLR is ≥ 7.7.

## Introduction

Since its initial outbreak in China in December 2019, SARS-CoV-2 soon spread to other countries, engulfing the world in the coronavirus 2019 (COVID-19) pandemic. Many variants of SARS-CoV-2, such as the Delta and Omicron variants, have been identified [1]. Previous studies reported that coinfection with other microorganisms, such as bacteria and fungi, can occur, albeit infrequently [2]. While there are some reports of COVID-19 and lower respiratory tract coinfection, there are few case reports or studies of COVID-19 with ­concomitant bacteraemia acquired in the community setting [3,4].

Patients with COVID-19 who have acquired bacteraemia as a nosocomial infection after hospitalization are sometimes encountered, but patients with COVID-19 and concomitant, community-acquired bacteraemia are rare. It is unclear how often COVID-19 co-occurs with community-acquired bacteraemia; a previous, retrospective study demonstrated that the rate was about 2.5% and that positive blood culture results were associated with increased mortality and ICU admission [4].

Patients with severe COVID-19 typically receive dexamethasone and an antiviral agent. Although these treatments are adequate for most patients, some patients have a bacterial coinfection requiring antibacterial therapy. Treatments for bacteraemia significantly differ from those for COVID-19.

Differentiating COVID-19 alone from COVID-19 with bacteraemia is challenging. Some of the risk factors and symptoms of bacteraemia and severe COVID-19 overlap; thus, having biomarkers capable of predicting bacteraemia in COVID-19 patients would be ideal. C-reactive protein (CRP) and the neutrophil-to-lymphocyte ratio (NLR) are common inflammatory markers, and some reports suggest that they can be useful in predicting mortality associated with COVID-19 [5]. Moreover, the NLR is thought to predict bacterial infection in COVID-19 as corroborated by a recent, retrospective study reporting that NLR ≥15 was associated with bacteraemia [4]. CRP is also considered to be a predictor of bacterial infection in COVID-19 [6], but its utility in predicting bacteraemia in COVID-19 is unknown. The present study therefore aimed to determine the cut-off value for CRP and NLR for predicting bacteraemia in patients with COVID-19.

## Methods

The present, retrospective cohort study was performed at Tokyo Metropolitan Tama Medical Centre, one of the largest tertiary care centres for patients with COVID-19 in Japan. The institutional ethical review board at the study centre approved this study (4-131), and the requirement for patient consent was waived because of its retrospective design.

Patients admitted to the hospital for COVID-19 treatment between 1 April 2020 and 30 October 2022 were included. Patients transferred from other hospitals and those whose CRP and/or neutrophil count was not measured at admission were excluded. Community-acquired bacteraemia was diagnosed if true bacteraemia diagnosed in patients *via* a blood culture performed within 72 h of admission. The definition of true bacteraemia followed that of the US Centres for Disease Control and Prevention/National Healthcare Safety Network for non-central line-associated blood stream infection [7]. Clinical data, including sex, age, oxygen administration, clinical outcomes, complete blood count, and CRP, were extracted from the electronic medical records of all the patients. Detailed clinical data on bacteraemia, such as the causative pathogen, infection source, and risk factors of bacteraemia (e.g. diabetes mellitus, liver cirrhosis, chronic lung disease, chronic kidney disease, rheumatic disease, active solid organ cancer, active hematological malignancy, HIV infection, injection drug use, alcohol abuse, history of solid organ transplantation, history of bone marrow transplantation, use of a glucocorticoid and/or immunosuppressive drug, endovascular prosthesis, and indwelling urinary catheter), were also collected.

Receiver operating characteristic (ROC) analysis using the Youden index method was performed to determine the cut-off value for CRP and NLR for community-acquired bacteraemia. Two-sided *p* < 0.05 was considered to indicate statistical significance, and all the analyses were done using JMP® version 11.2.0 (SAS Institute, Inc., Cary, North Carolina).

## Results

Between 1 April 2020 and 30 October 2022, 3347 patients were admitted for COVID-19 treatment, and 3031 patients were hospitalized from home or a nursing home. Forty-two patients were excluded owing to missing data on CRP and/or the neutrophil count at admission. The remaining cohort of 2989 patients comprised 1595 males (53.4%) and 1394 females (46.6%). The mean age of the cohort was 60.0 ± 22.0 years, and the mean CRP and NLR value was 4.88 ± 6.02 mg/dL and 5.20 ± 6.08, respectively. Of the 2989 patients, 1141 (38.2%), 130 (4.3%), 73 (2.4%), and 19 (0.64%) required standard oxygen therapy, high-flow nasal cannula, ventilation, and extracorporeal membrane oxygenation, respectively. Blood cultures were collected in 336 of the 2989 patients within 72 h of admission, and 19 patients received the diagnosis of community acquired bacteraemia.

Table 1 shows the demographic information, laboratory test results, causative pathogen, bacteraemia source, and outcome. The present cohort comprised 13 male and six female patients with a mean age of 70.1 ± 22.6 years. Eleven (57.9%) patients had one or more complications from among the risk factors of bacteraemia listed above, and eight (42.1%) patients had none. The bacteraemia source was identified in 12 (63.2%) patients but not in seven (36.8%) patients. Among the former, urinary tract infection and endovascular prosthesis-related bloodstream infection were the most common sources. The outcomes of the patients with COVID-19 and concomitant bacteraemia were poor, with five of the 19 (26.3%) patients dying during hospitalization; on the other hand, 124 (4.2%) of 2970 patients (including persons whose blood cultures were negative and those from whom no blood sample was collected) died during hospitalization.

**Table 1.** Summary of the characteristics of 19 patients with bacteremia.

Age, mean ± SD, years		70.1 ± 22.6
Female, *n* (%)		6 (31.6)
Comorbidities, *n* (%)	Diabetes mellitus	6 (31.6)
	Endovascular prosthesis	3 (15.8)
	Active solid organ cancer	2 (10.5)
	Chronic kidney disease	2 (10.5)
	Others^a^	5 (26.3)
	None	8 (42.1)
Organism, *n* (%)	*Staphylococcus aureus*	4 (21.1)
	*Escherichia. Coli*	3 (15.8)
	*Clostridium perfringens*	2 (10.5)
	*Streptococcus agalactiae*	2 (10.5)
	*Streptococcus pneumoniae*	2 (10.5)
	*Anaerobic Gram negative rods*	1 (5.26)
	*Bacillus subtilis*	1 (5.26)
	*Campylobacter ureolyticus*	1 (5.26)
	*Coagulase negative Staphylococcus*	1 (5.26)
	*Corynebacterium spp.*	1 (5.26)
	*Klebsiella pneumoniae*	1 (5.26)
	*Proteus mirabilis*	1 (5.26)
	*Streptococcus dysgalactiae*	1 (5.26)
Bacteremia Source, *n* (%)	UTI	3 (15.8)
	CRBSI	2 (10.5)
	Pneumonia	2 (10.5)
	Skin and soft tissue infection	2 (10.5)
	Others^b^	3 (15.8)
	Not identified	7 (36.8)
CRP, mean ± SD, mg/dL		17.5 ± 10.3
NLR, mean ± SD		22.7 ± 16.7
Outcome, *n* (%)	Died	5 (26.3)

CRP: C-reactive protein, NLR: neutrophil-to-lymphocyte ratio, UTI: urinary tract infection.

CRBSI: catheter-related bloodstream infection.

^a^ Others: alcohol abuse, chronic lung disease, immunosuppressive drug, rheumatic disease and use of glucocorticoid.

^b^ Others: biliary tract infection, enteritis and pacemaker infection.

ROC analysis demonstrated that the area under the curve (AUC) for the NLR was 0.880. Using the Youden index, NLR ≥ 7.7 was determined to be the cut-off value for bacteraemia. The sensitivity and specificity of the cut-off was 84.2% and 84.0%, respectively. The AUC for CRP was 0.850, and CRP ≥ 6.3 was determined to be the cut-off value for bacteraemia. The sensitivity and specificity of this cut-off was 89.5% and 73.3%, respectively.

## Discussion

The present study demonstrated that both CRP and NLR on admission can, as individual biomarkers, differentiate COVID-19 alone from COVID-19 with bacteraemia, and was the first to investigate the cut-off value of CRP and NLR for predicting bacteraemia in COVID-19. CRP and NLR are good predictors of disease severity and mortality in patients with COVID-19 [8,9] as well as of bacteraemia in patients without COVID-19 [10,11].

A previous, retrospective study reported that NLR ≥15 was associated with the co-occurrence of COVID-19 and bacteraemia [4]. The present study proposed a cut-off value of 7.7 for NLR, which is lower than in the study cited above. In the present study, 2.4% of patients required ventilation compared to 14% in the retrospective study, indicating a significant difference in disease severity, which may have influenced the variation in cut-off values. Setting the NLR cut-off value at 15 or above in the present study yielded a rather low sensitivity of 57.9% and a specificity of 95.3% for detecting bacteraemia. In clinical practice, it may be preferable to have a low threshold for obtaining blood culture samples.

COVID-19 can lead to immune system impairment during the disease course [12], which may in turn contribute to the co-occurrence of the disease with bacteraemia. However, the process is still unclear. It was important to differentiate COVID-19 alone from COVID-19 with bacteraemia because bacterial co-infection was associated with increased mortality. The complete blood count and CRP are commonly assessed on admission, and blood cultures should be obtained when CRP is ≥ 6.3 or NLR is ≥ 7.7.

## Data Availability

Data are stored at Tokyo Metropolitan Tama Medical Centre, and are available upon request.
